# Model for Glucagon Secretion by Pancreatic α-Cells

**DOI:** 10.1371/journal.pone.0032282

**Published:** 2012-03-07

**Authors:** Virginia González-Vélez, Geneviève Dupont, Amparo Gil, Alejandro González, Iván Quesada

**Affiliations:** 1 Departmento Ciencias Básicas, Universidad Autónoma Metropolitana Azcapotzalco, México City, México; 2 Unité de Chronobiologie Théorique, Université Libre de Bruxelles, Brussels, Belgium; 3 Departamento Matemática Aplicada y Ciencias de la Computación, Universidad de Cantabria, Santander, Cantabria, Spain; 4 Centro de Investigación Biomédica en Red de Diabetes y Enfermedades Metabólicas Asociadas (CIBERDEM), Instituto de Bioingeniería, Universidad Miguel Hernández, Elche, Spain; University of Bremen, Germany

## Abstract

Glucagon hormone is synthesized and released by pancreatic α-cells, one of the islet-cell types. This hormone, along with insulin, maintains blood glucose levels within the physiological range. Glucose stimulates glucagon release at low concentrations (hypoglycemia). However, the mechanisms involved in this secretion are still not completely clear. Here, using experimental calcium time series obtained in mouse pancreatic islets at low and high glucose conditions, we propose a glucagon secretion model for α-cells. Our model takes into account that the resupply of releasable granules is not only controlled by cytoplasmic 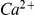, as in other neuroendocrine and endocrine cells, but also by the level of extracellular glucose. We found that, although calcium oscillations are highly variable, the average secretion rates predicted by the model fall into the range of values reported in the literature, for both stimulated and non-stimulated conditions. For low glucose levels, the model predicts that there would be a well-controlled number of releasable granules refilled slowly from a large reserve pool, probably to ensure a secretion rate that could last for several minutes. Studying the α-cell response to the addition of insulin at low glucose, we observe that the presence of insulin reduces glucagon release by decreasing the islet 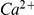 level. This observation is in line with previous work reporting that 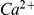 dynamics, mainly frequency, is altered by insulin [1]. Thus, the present results emphasize the main role played by 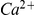 and glucose in the control of glucagon secretion by α-cells. Our modeling approach also shows that calcium oscillations potentiate glucagon secretion as compared to constant levels of this cellular messenger. Altogether, the model sheds new light on the subcellular mechanisms involved in α-cell exocytosis, and provides a quantitative predictive tool for studying glucagon secretion modulators in physiological and pathological conditions.

## Introduction

Pancreatic islets of Langerhans, which play a crucial role in glucose homeostasis, are mainly formed by three types of exocytotic cells (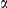 , 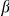 and 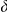). 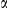-cells represent 15 to 20% of islet cells and secrete glucagon in response to decreased blood glucose levels. Glucagon in turn stimulates glucose production by the liver. In contrast, insulin is secreted by pancreatic 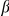-cells when plasma glucose concentrations are elevated, leading to the insulin-dependent glucose uptake by peripheral tissues. Impaired glucagon secretion has been associated to hyperglycaemic periods in diabetic patients. Despite their importance, 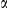-cells are much less studied than 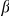-cells most probably because of several technical limitations [2]. More surprisingly, as far as we know, there is no modeling study devoted to the molecular mechanisms regulating glucagon secretion by this cell type, although we have reported a modeling study of electrical currents related to glucagon secretion [3]. In contrast, there have been several theoretical works devoted to the secretion of insulin by pancreatic 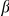-cells (for example [4]–[6]).

Experimentally, measurements of glucagon secretion are commonly obtained by incubating pancreatic islets in media with low glucose concentrations for several minutes. Under low-glucose levels (stimulated), the total amount of secreted glucagon in mice ranges from 30 to 40 picograms per islet (pg/islet) in one hour, and this amount is approximately twice the basal (non-stimulated) secretion that is seen at the high glucose concentrations typically used in these experiments [7]–[10]. These quantities are inferred from measurements of the accumulated glucagon for a one hour period from a large number of islets, each mouse islet containing on average, 400
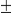
200 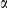-cells [2]. Such an stimulated glucagon secretion could be produced by a constant rate between 0.5 and 1 pg/islet (or fF) per minute, as reported in one experimental work [7].

Glucagon is released by 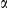-cells through the 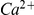 -dependent exocytosis of secretory granules. The mechanism responsible for the modulation of intracellular 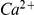 by extracellular glucose levels is still a matter of debate and has been ascribed either to a direct glucose effect, or to a paracrine effect involving 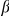-cells [11]. The direct effect could be managed by the 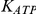 channels present in 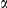-cells which are glucose-sensitive [10], or by a glucose-induced alteration of 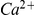 -storing mechanisms [9]. Although the signal-transduction pathway relating low glucose levels to 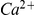 increase remains to be further clarified, these 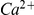 rises have been well characterized. Opposite to what happens in 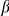-cells, that all of them undergo synchronized calcium oscillations upon stimulation with high glucose levels [12], about 30% of the total islet 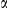-cells exhibit oscillations when exposed to low-glucose levels [13]. These oscillations are asynchronous and highly irregular. Oscillation frequency can go from 0.1 to 0.7 per minute at 3 mM glucose [12], [14], [15], up to 0.5 to 1 per minute at 0.5 mM glucose [16]. The amplitude of these oscillations runs from basal 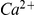 levels (near 100 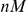) up to two to five times this value [13], [14], [17]. This spontaneous behaviour has been widely observed in isolated 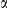-cells [13], [14], in clonal cell lines [16], [18], and in intact islets [12].

Exocytosis by 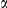-cells in response to electrical stimulation has been well characterized. In these experiments, the secretory response is inferred from the associated increments in cell membrane capacitance due to granule fusion [7], [10], [19]–[21]. In contrast, there are few reports of experiments performed on islets, or on isolated 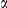-cells, that relate 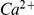 oscillations induced by low glucose concentrations to glucagon release, in the presence of various pharmacological modulators [8], [13], [15]. Both stimulatory protocols (electrical or low-glucose), however, induce highly different levels of secretion. Under electrical protocols, the release evoked by a millisecond depolarization reaches rates up to 150 granules per second [20], whereas the secretion rates attained under low-glucose stimulation are in the order of 0.5 granules per minute, equal to 1 fF per minute considering an increase of membrane capacitance of 2 fF per granule as stated in [19]. Moreover, experimental data about dynamic secretion rates under low-glucose stimulation are scarce [7]. Therefore, there is a clear need to explore glucagon secretion dynamics using modeling techniques.

In this work, we propose a secretion model for 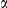-cells based on experimental data of 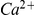 recorded in intact islets, in response to low and high glucose. As the signaling pathway linking the level of glucose and the concentration of intracellular 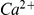 remains unclear, it is not considered explicitly in the model. Instead, the different glucose concentrations are taken into account in the model by different 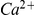 oscillatory patterns obtained experimentally at these glucose concentrations. Such 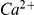 time series are taken as input to the simulations, and they feed our model for 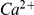 -mediated secretion. Our model incorporates that the resupply of releasable granules is not only controlled by cytoplasmic 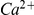 , as in other neuroendocrine and endocrine cells [22], [23], but also by the level of extracellular glucose. The output of the model, i.e. the amount of glucagon secreted, is compared to secretion levels induced by the same glucose concentrations, which have been measured in our laboratory and/or are available in the literature. We have also obtained 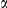-cell 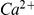 records in the presence of insulin at low glucose, and used the model to predict the effect of this hormone on glucagon secretion.

## Methods

### Experiments

All protocols were conducted following the regulations approved by the Animal Care Committee of the Universidad Miguel Hernández (approval ID: IB-IQM-001-10), according to national and European policies about ethics in animal research. Swiss albino OF1 mice (8–10 weeks old) were sacrificed by cervical dislocation and pancreatic islets were then isolated by collagenase digestion as previously described [8]. Single islets were loaded with 5 mM of the acetoxymethyl form of the 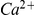 probe Fluo-4 (Molecular Probes; Leiden, The Netherlands) for at least 1 hour at room temperature in a medium containing (mM): NaCl, 115; NaHCO3, 10; KCl, 5; MgCl2, 1.1; NaH2PO4, 1.2; CaCl2, 2.5; HEPES, 25; bovine serum albumin, 1%; D-glucose, 5 mM, pH = 7.4. All experiments were carried out at 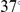
C.

For imaging experiments, islets were placed on a perfusion chamber mounted on the microscope stage and attached onto poly-L-lysine treated coverslips for 10 min before commencing the experiments. Islets were then perfused at a rate of 1.5 ml/min with a modified Ringer solution containing (mM): 120 NaCl, 5 KCl, 25 NaHCO3, 1.1 MgCl2 and 2.5 CaCl2; pH 7.4, gassed with 95% O2 and 5% CO2. 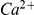 signals were monitored in individual cells within the islets using a Zeiss LSM 510 laser confocal microscope equipped with a 40× oil immersion objective (numerical aperture = 1.3). The system configuration was set to excite the 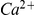 probe at 488 nm and collect the emission with a bandpass filter at 505–530 nm from an optical section of 8 mm. Images were collected at 2 s intervals. Temporal series were treated with a low pass filter and processed using the digital image software of the confocal microscope. Pancreatic 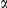-cells were functionally identified by their characteristic oscillatory 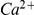 signal at 0.5 mM glucose [8], [12], [24]. Calcium oscillations were recorded for 10 minutes at 0.5 mM glucose (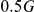), which stimulate 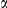 cells, followed by a subsequent period of 15 minutes at 11 mM glucose (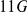) which inhibits the oscillatory calcium activity in these cells.

**Figure 1:**
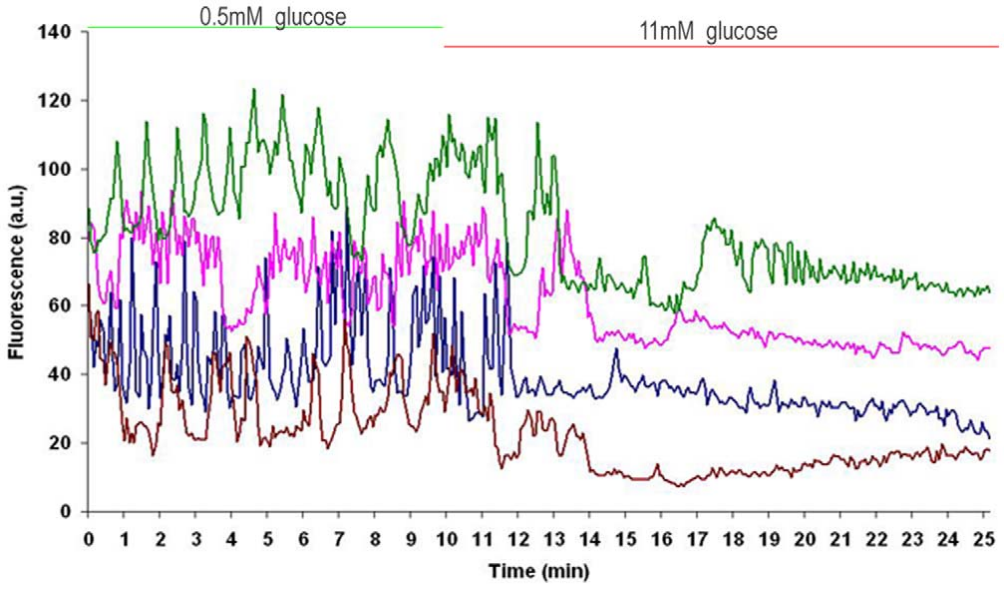
Four experimental 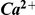
 **oscillations from** 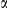 **-cells in intact islets.** Islets are perfused with a solution containing 0.5 mM glucose for the first 10 minutes, and then, solution is changed to one containing 11 mM glucose for the following 15 minutes. Oscillations, triggered by low glucose concentrations, are highly irregular. The level of 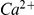 is indicated in arbitrary fluorescence units.

Following these methods, 2 different sets of 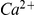 data were obtained. The first set is made of 14 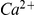 oscillation traces, lasting 25 minutes. These 25-minute records consist of 10 minutes at 0.5 mM glucose (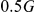), which stimulate 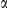-cells, followed by 15 minutes at 11 mM glucose (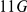), which inhibits the oscillatory calcium activity in these cells. The second set is composed by 23 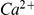 oscillation traces, lasting 16 minutes (6 min at 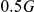 followed by 10 min at 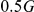
+4 nM insulin). In both sets each record corresponds to a single 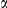-cell found in the first layer of an islet, and cells were issued from three islets for each set. In Figure 1, some examples of the recorded 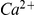 oscillations of the first set are shown. Note that the first five minutes at 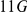 correspond to a transition period in which glucose levels are changing in the bath, so they are not used for the modeling work.

### Model description

**Figure 2:**
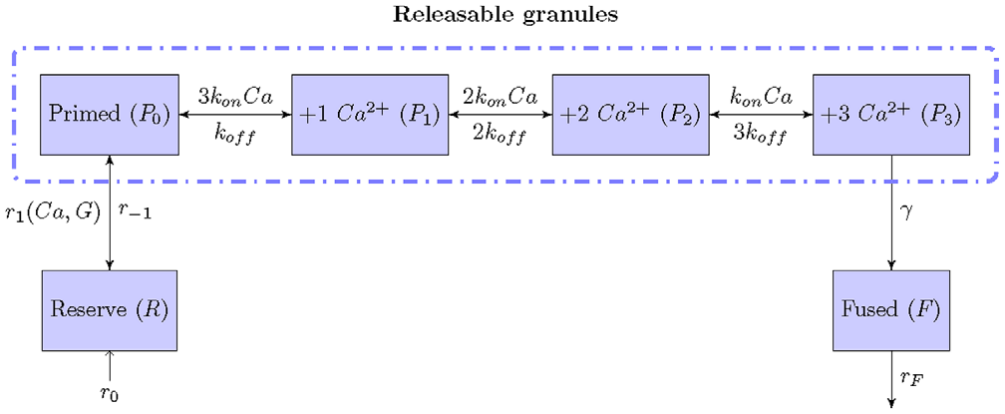
Stages proposed for glucagon secretion. Granules coming from the Reserve pool (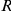) resupply the Primed pool (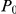) and then, upon binding 3 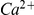 ions, granules become Fused (pool 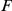). The Releasable stage includes all primed granules with 0 to 3 bound 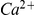 . 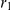 and 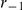 are the forward and backward resupply rates, respectively. 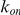 and 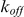 are the binding and unbinding rates, respectively, associated to the 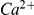 -sensor. 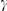 stands for the fusion rate that manages the last non-reversible step of exocytosis. 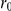 and 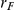 represent the rates of granules formation and granule release, respectively.

The kinetic model for glucagon secretion, which describes 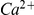 -triggered exocytosis in 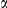-cells, is schematized in Figure 2. It is based on previously developed models describing secretion in chromaffin cells [25] and in pancreatic 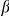-cells [5], and it has been adapted to take into account observed specificities of pancreatic 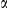-cells. Two types of secretory granules are considered in the model. The first type constitutes the so-called ‘reserve pool’ (pool 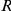) located in the cytoplasm. These granules are susceptible to be mobilized towards the membrane and to undergo a maturation process. These transformations are called ‘docking’ and ‘priming’, respectively, and are grouped in a single step in the model. After this transition, granules are ‘ready to be released’ and are named ‘primed’ granules (pool 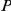). In addition, 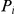 stands for the pool of primed granules bound to 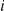 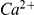 ions, and 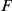 is the pool of fused granules. Usually, fusion to the membrane and secretion occur in response to a considerable 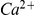 increase in the microdomain located just below the plasma membrane [22]. Therefore, we consider that 3 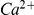 ions are necessary to trigger granule fusion, similarly to what has been suggested for 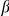-cells [5], [6].

The variables of the model are the number of granules in each of the 6 states schematized in Figure 2. Transition from the reserve pool to the primed pool is supposed to be a reversible process. Priming is supposed to be activated by cytosolic 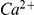 as in other cell types [5], [25]. Specific to 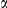-cells is the fact that resupply is also somehow sensitive to the extracellular glucose level, independently from 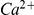 . It has indeed been demonstrated that an increase in extracellular glucose from 0 to 20 mM provokes a 3.3 fold rise in glucagon secretion evoked by a train of depolarizations in isolated 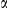-cells [26]. Moreover, Andersson et al. [27] have recently used electron microscopy to show that the number of granules in close vicinity to the plasma membrane is larger at high glucose than at low glucose. As the molecular mechanism by which external glucose activates granule resupply is far from being fully elucidated, and since we have data recorded at two glucose concentrations, we simply assume in the model that the rate of resupply is a linear function of extracellular glucose. Thus, the rate of change of the number of granules in the reserve pool 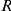 can be written as:
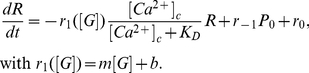
(1)


In this equation, 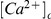 represents the concentration of 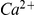 in the cytosol, 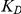, the value of 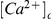 leading to half activation of resupply by 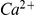 , 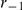, the rate constant characterizing the transition of primed granules to the reserve one, 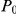, the number of granules in the primed state without bound 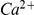 , and 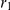, the resupply rate at infinite 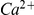 concentration. The dependence of this rate on the external glucose concentration (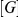) is taken as a linear function characterized by parameters 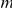 and 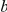. The term 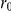 stands for the rate of granule formation. This term needs to be included in the model in order to allow for the existence of a steady state. Further steps represent 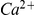 binding on docked granules. These reversible binding reactions are described by the mass action law. Thus,
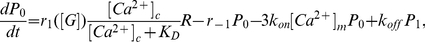
(2)

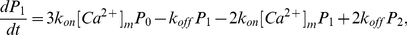
(3)

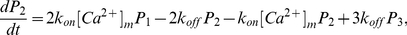
(4)

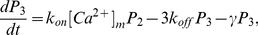
(5)

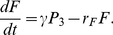
(6)


In equations (2)–(6), 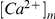 represents the concentration of 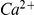 just beneath the plasma membrane, where granules are docked; 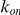 and 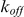 stand for the rates of 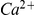 binding and dissociation, respectively; and 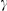 is the rate constant for granule fusion. There might be a delay between fusion and glucagon release, as found for insulin secretion [23]. This delay is introduced through the 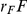 term, which is also necessary to get a steady state in the model, as 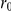. In the Results section, accumulated secretion per hour or per minute is computed as 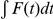 over the corresponding time interval.

Since experimentally obtained oscillations reflect cytoplasmic 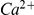 concentration (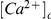), we estimated the corresponding dynamic values for the local 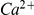 concentration just below the 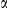-cell membrane (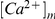). In 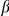-cells, it has been suggested than secretory granules are exposed to very high levels of local calcium (up to 10 times higher than in the cytosol) [28]. No data are available for 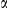-cells probably due to the small size of these cells. Thus, considering that granule diameter is between 260 and 290 nm [19] and, that granules are located just below the plasma membrane [7], we performed simulations to estimate local 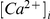 beneath the plasma membrane. We used our previously published model that estimates intracellular 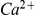 in concentric layers of specific thickness for a spherical cell considering buffered diffusion [29]; to this model, we added a 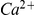 -extrusion pump [30] and a compensating leak flux. Leak flux was adjusted to obtain a steady-state 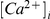 close to 0.1 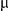
M. Assuming that an 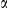-cell could be approximated by a spherical cell of 5.3 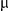
m radius [19] with a large nucleus (60% of the cytoplasm), we obtained that 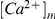 to 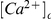 ratio ranges between 1.26 to 1.29 depending on the spatial extent considered for the layer beneath the membrane (from 10 to 300 nm). For all subsequent simulations we have taken 100 nm, for which the ratio equals 1.28. Parameter values used to estimate membrane 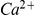 concentration (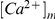) are given in Table 1.

**Table 1:** Parameters to estimate membrane 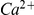 concentration.

Parameter	Value	Source
Cell radius	5.3 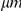	[19]
Layer thickness	100 nm	Chosen
Endogenous buffer	500 mM with 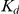 = 10 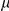 M	[30]
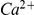 -extrusion pump	2 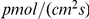 with 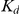 = 0.83 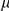 M	[30]
Leak flux	21.9 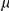 M/s	Fitted

### Parameter estimation

Using 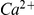 concentrations recorded at 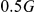 and 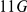 as inputs to the secretion model, we estimate the adequate values for parameters 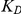, 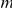, 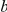, 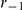, 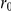, 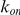, 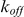, 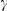 and 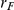. We took as starting points those values used in previously developed models for secretion in chromaffin cells [25] and in pancreatic 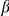-cells [5], and adapted them using two different constraints:

1. *To reproduce steady-state values of both the total number of granules and the basal secretion rate.* Total number of granules for each 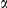-cell (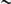
5800) was estimated taking a mean cell radius of 5.3 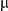
m [19], that gives an average 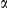-cell volume of 623.6 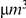, and considering a granule density of 9.3 granules/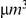 [19]. This granule population is in the range of the 4400 granules observed by [27]. Basal secretion rate at high glucose (10 mM) has been estimated in 16 to 20 pg/islet/hr (0.25 to 0.33 pg/islet/min) [7], [8]. These data were used to fix parameters 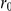 and 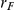.
2. *To predict the experimentally reported secretion in response to* 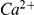 *oscillations monitored in* 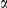 *-cells of mice, in the presence of low glucose.* At low glucose (0.5 and 1 mM), secretion is in the range 30–60 pg/islet/hr (equivalent to 0.15–0.30 pg/cell/hr) [7]–[10], which is twice the secretion rate at high glucose. To reproduce this rate, we fitted parameters 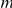, 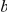, 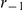 and 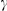.

The remaining parameters (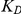,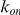,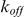) were taken from the literature [5], [25]. Fitting was performed manually. It was tested that results are not sensitive to slight variations of the parameter values. The final set of parameter values as well as those values used to quantify secretion are shown in Table 2. The granule equivalence in femtograms (fg) was obtained considering that one islet contains about 2 nanograms of glucagon [31] and a minimum of 200 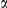-cells [2], so each cell contains, at most, 10 picograms of glucagon. Considering that an 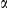-cell possesses 5800 granules, the granule content would be 1.72 fg of glucagon, then for simulations, we are taking a round value of 2 fg per granule.

**Table 2:** Parameters used to simulate secretion dynamics.

Parameter	Value	Reference
*Secretion model*		
Glucose function	m = 3; b = 18.5	Fitted
Glucose concentration	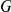 = 0.5 and 11 mM	Experiments
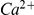 affinity of resupply	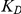 = 2.3 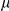 M	CC [25], 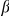 C [5]
Backward resupply rate	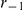 = 2.1 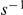	Fitted
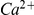 -binding rate	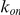 = 0.5 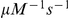	CC [25]
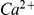 -dissociation rate	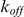 = 4 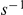	CC[25]
Fusion rate	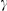 = 0.011 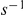	Fitted
Granule formation rate	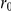 = 0.0001 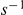	Fitted
Granule release rate	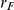 = 0.01 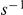	Fitted
*Other values*		
Cellular volume	623.6 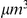	Computed
Granule density	9.3 granules 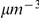	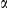 C [19]
Granule equivalence	2 fg	Computed

CC = chromaffin cells, 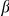
C = 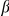-cells, 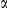
C = 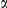-cells.

### Simulations

The purpose of the secretion model is to quantitatively predict the level of glucagon secretion induced by real 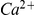 oscillations in 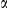-cells from intact islets at different conditions. Two sets of 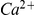 data were supplied from experiments: One set was composed of 14 records (14 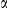-cells) coming from 3 islets going from 0.5 mM to 11 mM glucose, and the other set was made of 23 records (23 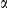-cells) coming from 3 other islets at 0.5 mM glucose, with and without exogenous insulin (4 nM). These records of 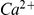 oscillations are in arbitrary fluorescence units so, to fit parameters in molar units, we seek for a reliable equivalence. Our data have not been calibrated but we simply compared them to those measured by other groups using similar techniques [13], [15]. We indeed mainly focus on the 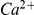 dynamics for which accurate calibrations of the absolute 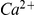 levels are not necessary. We thus assume that one fluorescent unit approximately corresponds to two nanoMolar units of 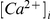 . On the basis of this empirical procedure, the values of cytoplasmic calcium range between 30 to 400 nM, in agreement with values reported in the works mentioned above.

Experimental 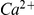 data were used as input to the secretion model defined by equations (2)–(6). These simulations correspond to the dynamics of secretion during either 20 minutes or 1 hour. In both types of simulations, secretion computed for each cell was shown as the individual secretion rate, and the predicted average secretion rate was calculated as the average of all cells in an islet. On the one hand, the 20-minute simulations were performed using the experimental 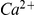 records, taking 10 minutes in one condition (low glucose) followed by 10 minutes in the other condition (high glucose or presence of insulin). These dynamic simulations brought out differences between individual and average (islet) responses. Moreover, these simulations were also useful to appreciate the time needed to reach a steady-state response. On the other hand, one-hour simulations used as inputs the same 10-minute records repeated six times. This methodology was thought to make comparable predictions of glucagon secretion, since in experiments accumulated secretion is generally reported as pico- or femtograms per islet in one hour.

To illustrate the applicability of the model as a predictive tool, we have studied the effect of insulin on glucagon secretion by performing the same kind of simulations (20-minute and one-hour) using experimental 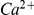 records. These simulations allowed us to compare the individual and the islet response to insulin and glucose. Our hypothesis was to explore if the experimentally reported effect of insulin on glucagon secretion may be entirely ascribable to the action of this hormone on the individual and the islet 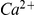 levels.

All simulations were performed using the parameter values listed in Table 2.

## Results

### Dynamics of secretion induced by low- and high-glucose periods

**Figure 3:**
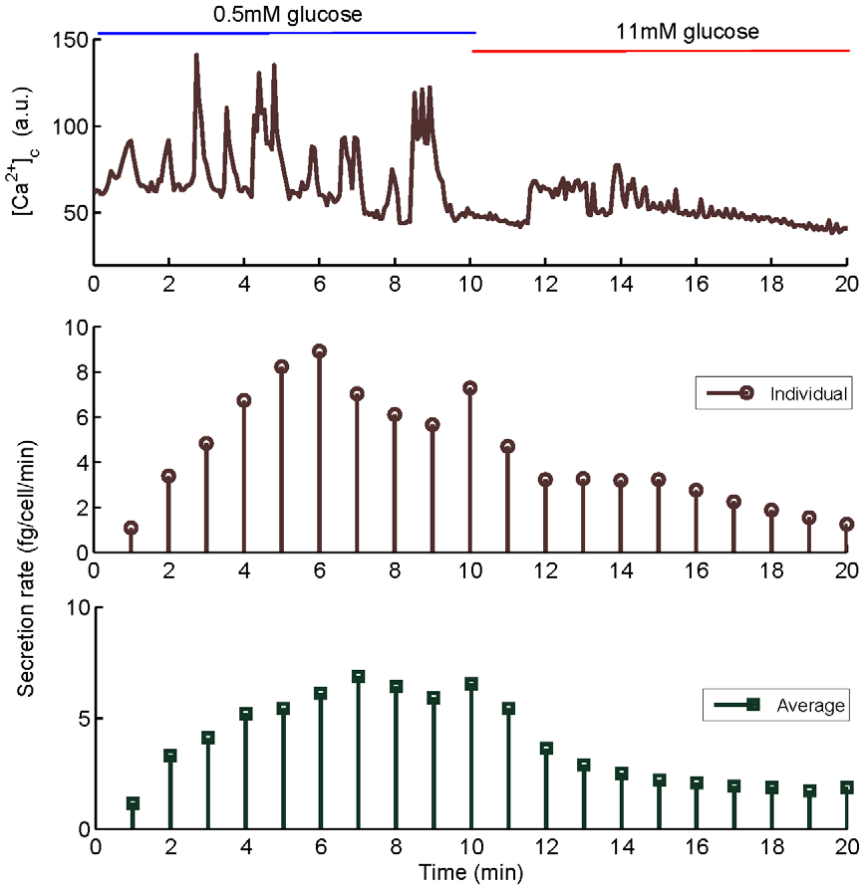
Example of 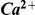
 **and secretion dynamics in a 20-minute period, going from low (** 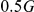 **) to high glucose (** 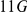 **).** The 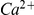 time-series are shown in arbitrary units (top), while simulated individual (middle) and average (bottom) secretion rates are in fg per cell per minute. Secretion is computed as the numerical integral of 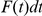, taking into account that 1 granule corresponds to 2 fg of glucagon. Notice that individual and average secretory responses have the same trends. All individual responses to low and high glucose are summarized in Figure 4.

In Figure 3 we show an example of a dynamic simulation of secretion obtained with the secretion model defined by equations (1)–(6). The top panel shows the 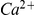 time series used as input to the model, the middle panel shows the individual secretion induced by this 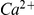 time series, and the bottom panel represents the average secretion of the 14 cells. Cytoplasmic 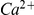 oscillations induce a secretion rate per minute that seems to have a delayed maximal response to low glucose, as reported in [7]. This delay corresponds to the time needed by granules to become releasable, as about half granules are in the reserve pool at resting 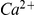 . As expected, steady-state (after five minutes) secretion rate at low-glucose is greater than at high-glucose for the whole set of cells.

### Steady state distribution of granules

We have estimated the population of releasable granules at steady-state for both glucose conditions; this population includes all primed granules belonging to the releasable stage considered in our model (Figure 2). The population predicted by the model, at high-glucose, was 49% in the Primed pool (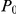) plus 3% in the +1 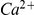 pool (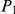). The resultant percentage (52%) compares well with the 55% found as the population of submembrane granules located in the first 300 nm below the plasma membrane in [7], suggesting that releasable granules in non-stimulated alpha cells would be located between this distance as in neuroendocrine cells [22]. In contrast, the model predicts that at low-glucose, 28% of granules are in the Primed pool (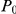) and 2% are in the +1 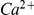 pool (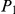), giving 30% of releasable granules. In both cases, the predicted populations for 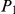 match well with estimations of the readily-releasable pool (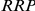) in 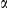-cells (1 to 2%) [19], [28], which are larger than in 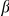-cells (0.2 to 1%) [23]. Moreover, our predictions are in good agreement with the reported observation that there are more docked granules at high glucose conditions than at low glucose [27]. On the other hand, having a larger releasable pool at high glucose is supported by the fact that under this condition, ATP levels should be higher so the priming step is favored since it is ATP-dependent, as considered for 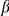-cells [23].

### High-glucose and low-glucose secretion in one hour

**Figure 4:**
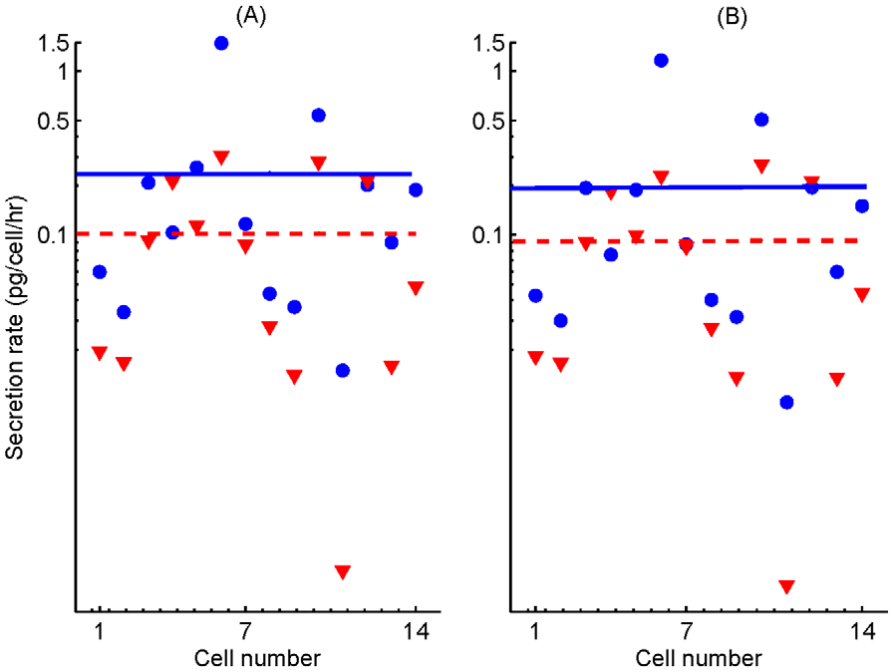
Individual cell secretion rates induced by 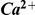
 **oscillations (A), and by constant** 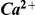 **levels (B).** The constant levels used for simulations correspond to the individual mean values of 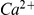 oscillations. Secretion rates in pg per cell per hour predicted at 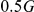 (dots) and 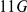 (triangles) are shown. Secretion in one hour is computed as the numerical integral of 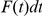, taking into account that 1 granule corresponds to 2 fg of glucagon. Lines indicate average secretion rates of the ensemble at low (blue) and high glucose (red). In agreement with experimental measurements, the model predicts that mean glucagon secretion is reduced at high glucose.

Individual secretion rates induced by each of the 14 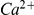 time-series at 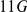 and 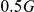 were predicted using one-hour files, as explained in the Methods section. Then, mean secretion of the ensemble in one hour was calculated to compare it to experimental secretion. In Figure 4(A), we show the resulting individual secretion rate per hour at 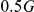 (blue dots) and 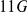 (red triangles), as well as the average of all cells for low (0.242 pg/cell/hour, blue line) and high (0.103 pg/cell/hour, red line) glucose. These values indicate a high-glucose-induced reduction in secretion of 57%. Accumulated secretions in 1 hour predicted by the model are 20.6 and 48.3 pg/islet at high and low glucose concentrations, respectively, taking 200 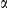-cells per islet; these accumulated values are in the range of experimental measurements [7], [8], [10], [32]. From this figure, we also predict that eight cells out of 14 (57%) clearly secrete more glucagon at 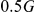 than at 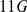, 5 cells (36%) secrete almost equal at both levels, and one cell (7%) secretes more glucagon at 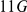 than at 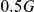 (see Table 3). These results agree with reported ideas that 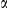-cells work in an asynchronous manner, and with the known unequal participation of each cell to whole-islet glucagon secretion [24].

**Table 3:** Distribution of individual 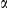-cell secretion.

		Percentage of cells	
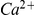 dynamics	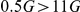	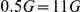	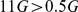
Oscillations	57%	36%	7%
Constant	50%	36%	14%

### Secretion induced by constant 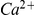 levels

In order to explore the impact of intracellular 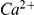 oscillations on glucagon secretion from a theoretical point of view, we also tested the secretion predicted by our model for a constant 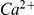 level equal to the mean value obtained for each cell at a given glucose concentration. This question is physiologically relevant given that only 30% of total islet 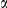-cells exhibit oscillating calcium levels [13]. The effect of an oscillatory 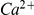 dynamics on various cellular responses has indeed been studied by modeling in other cell types. For example, it was predicted that 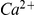 oscillations in liver cells can potentiate glycogenolysis in response to low levels of stimulation [33]. In other systems, where 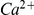 -calmodulin kinase II is involved, the level of physiological response is encoded in the frequency of 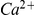 oscillations [34]. In this sense, results about frequency, shown in next section, aim at testing if such type of potentiation is present in 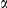-cells.

In Figure 4(B) we show the individual secretion rates obtained for mean 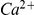 levels. In this case, the predictions of the model are: seven cells out of fourteen (50%) clearly secrete more glucagon at 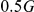 than at 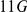, five cells (36%) secrete almost equal at both levels, and two cells (14%) secrete more glucagon at 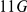 than at 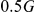. Moreover, average secretion rate at low glucose (0.198 pg/cell/hr) is still above the rate at high glucose (0.093 pg/cell/hr), i.e. there is still 53% of high-glucose-induced reduction on secretion. In contrast, for low glucose concentration -which most of the time induce 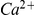 oscillations- the secretion rate is higher for an oscillatory 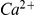 signal (0.241 pg/cell/hour) than for a constant 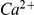 signal with the same average (0.198 pg/cell/hour); that is, oscillations induce 21% more secretion. Moreover, the distribution of individual participation in total islet release is different for a constant than for an oscillatory calcium level, as summarized in Table 3. The model thus predicts that upon low-glucose stimulation, 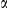-cells that display 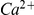 oscillations secrete more glucagon than those exhibiting a steady increase in 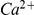 with the same average.

### Effect of oscillation frequency and mean 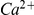 levels on secretion

**Figure 5:**
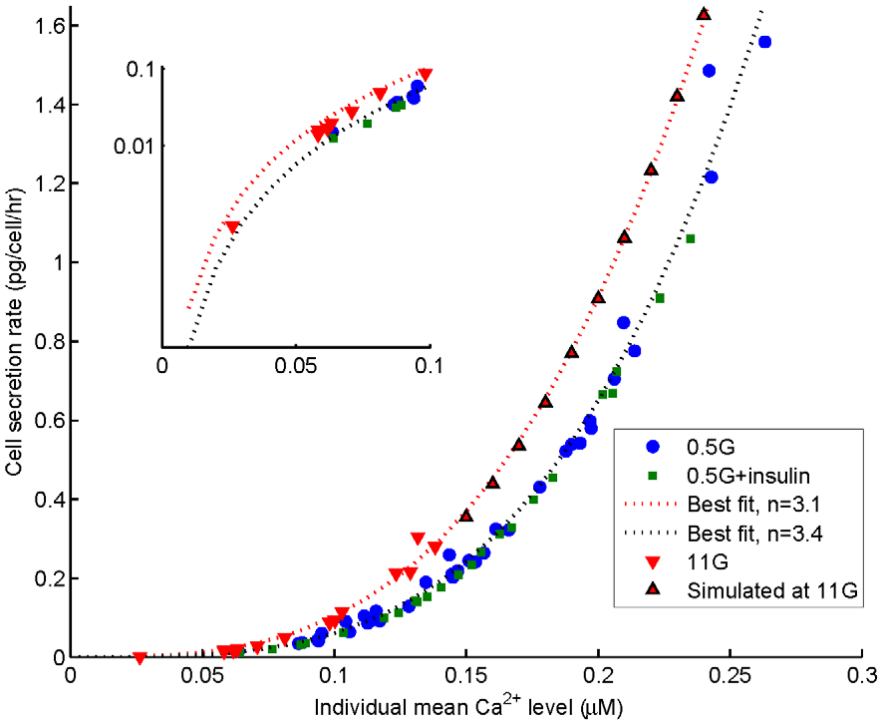
Cell secretion rates as a function of individual mean 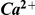
 **level.** Secretion rates are plotted against the mean 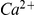 level of each cell, for different experimental and simulated conditions (see plot legend). Secretion in one hour is computed as the numerical integral of 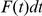, taking into account that 1 granule corresponds to 2 fg of glucagon. Best-fit curves for data sets are power functions with correlation coefficients 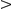
0.9. Notice that for a given value of individual mean 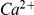 , the presence of low glucose (dots) induces a lower secretion rate than the high glucose (triangles), because of the glucose-dependence of granule resupply. The inset shows the behaviour of secretion rates at the lowest 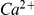 range, indicating that for a mean 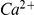 level of 0.1 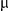
M, the model predicts a basal rate about 0.1 pg/cell/hr (20–40 pg/islet/hr) in agreement with experimental observations [7]–[10].

The results shown in the preceding sections point toward the fact that oscillations favor secretion. To understand the reasons of this increase, we have first investigated the relationship between glucagon secretion and the frequency of 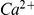 oscillations. Frequencies are between 0.3 to 1.6 per minute at 0.5 mM glucose, with a mean value of 0.87 per minute, as in previous experiments [16]. However, we found no correlation between secretion rate and frequency (not shown). This suggests the absence of frequency coding, which could be expected given the high level of irregularity of 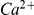 oscillations in 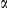-cells. Therefore, in Figure 5, computed rates of secretion are shown as a function of individual mean 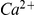 level, considered as constant throughout the simulation. Red triangles and blue dots indicate secretion rates obtained with constant 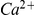 levels corresponding to experimental records at 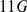 and 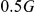, respectively. We also represent in this figure secretion rates obtained at arbitrary 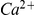 concentrations (red upward triangles), and at concentrations measured in the presence of insulin (green squares) as discussed in next section. The curves obtained at high and low glucose are different because resupply depends on glucose concentration, hence the secretion rates. Interestingly, these curves are steep, which provides a clue to understand why oscillations favor secretion. Indeed, because of this highly non-linear relationship between mean 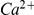 and secretion rate, a small increase in 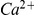 concentration produces a large increase in secretion rate; thus, average secretion during oscillations is larger than the secretion obtained over the same period of time with constant 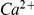 . Such a type of oscillation-induced potentiation of a 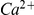 -activated response, has been demonstrated experimentally for gene expression [35].

### Prediction of glucagon secretion in the presence of insulin

**Figure 6:**
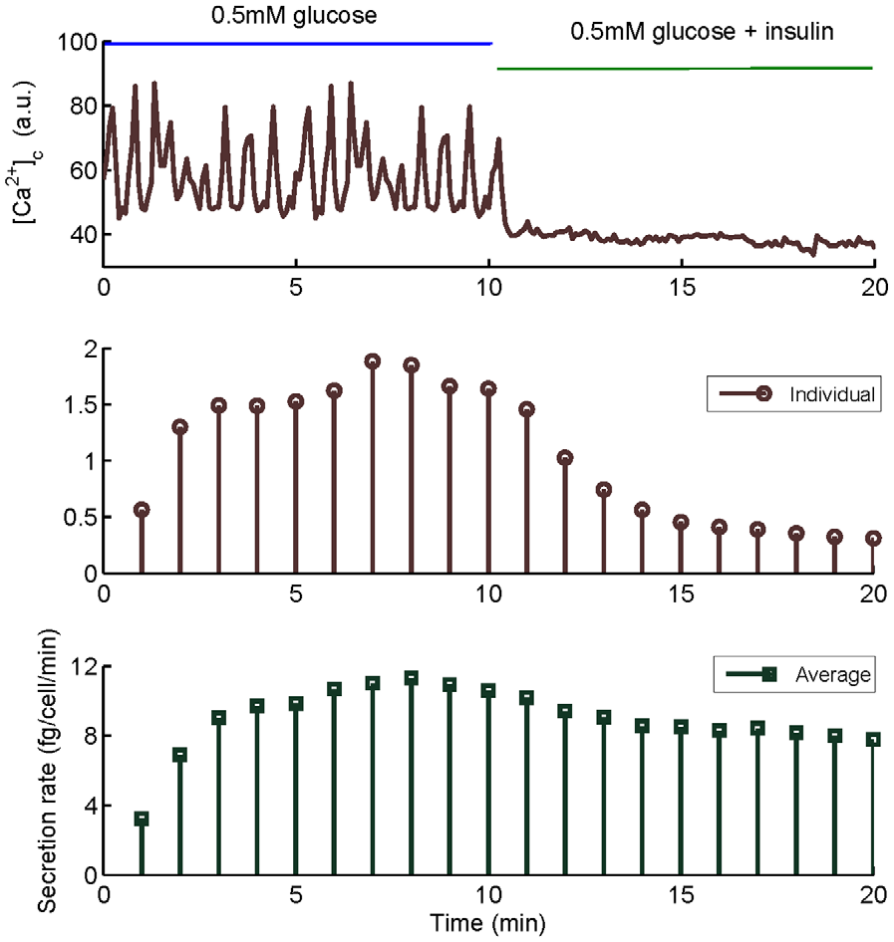
Example of 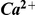
 **and secretion dynamics in a 20-minute period at low glucose (** 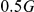 **), before and after the addition of 4 nM insulin.** The 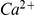 time-series in arbitrary units (top), as well as the simulated individual (middle) and average (bottom) secretion rates are shown in fg per cell per minute. Secretion is computed as the numerical integral of 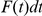, taking into account that 1 granule corresponds to 2 fg of glucagon. Notice that individual and average secretory responses have the same trends.

**Figure 7:**
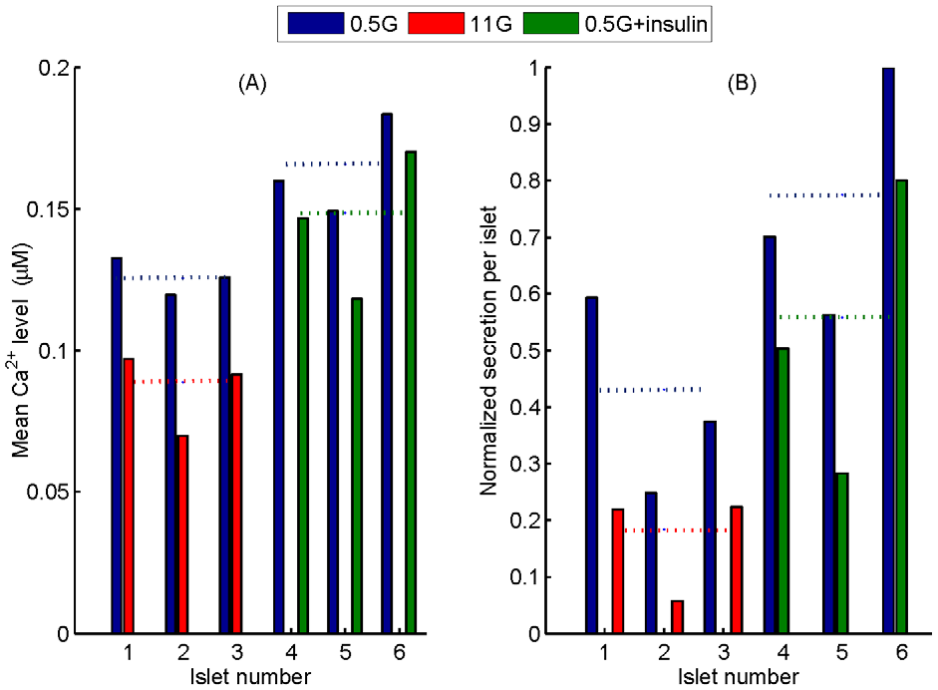
Effect of insulin and high glucose on mean 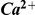
 **level and glucagon secretion for the six studied islets.** (A) Reduction of the mean 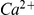 level due to the elevation of glucose in islets 1–3 (blue and red bars in the left), compared to the reduction induced by insulin at low glucose in islets 4–6 (blue and green bars in the right). Average 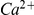 levels are indicated with dashed lines. (B) Glucagon inhibition induced by increasing extracellular glucose in islets 1–3 (blue and red bars in the left), compared to the inhibition induced by the presence of insulin in islets 4–6 (blue and green bars in the right). Secretion is normalized to the largest value to emphasize differences. Average secretions are marked with dashed lines.

It is indeed known that in physiological conditions, insulin and glucose both modulate glucagon release in 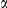-cells, although the mechanisms involved in this regulation are still under debate [11]. Insulin inhibits glucagon secretion, but this inhibition remains poorly understood [36]. In Figure 6 we show a dynamic simulation for these conditions, where the top panel shows the 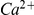 time series used as input to the model, the middle panel shows the individual secretion induced by this 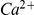 time series, and the bottom panel represents the average secretion of the 23 cells. For these new simulations, we found an average secretion rate at 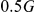 of 0.435 pg/cell/hour, different than the value obtained with the previous set of 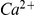 data in the same conditions (0.241 pg/cell/hour). However, this difference is of the order of variability in secretion among different islets (see Figure 7(B)). In the presence of insulin the model predicts that average secretion falls to 0.313 pg/cell/hour. This predicted 30% reduction is in the order of previously reported insulin-induced inhibitions at low glucose, for different concentrations of insulin (17 to 100 nM) [1], [26]. Then, from our results at low glucose, glucagon inhibition due to 4 nM insulin is smaller than to high glucose. However, it is probable that higher insulin concentrations would be more potent.

In Figure 7(A) we show the mean 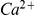 levels managed by each of the six islets that have been considered in the present study (3 islets under low and high glucose, and 3 islets under low glucose with and without insulin); all these 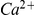 values are computed from experimental records. In Figure 7(B) we compare the inhibition of secretion induced by glucose elevation and by insulin. We have found that both situations result in the inhibition of secretion, and that this effect is associated to a reduction of the islet 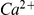 level; then, a smaller 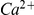 reduction induces a smaller inhibition, as in the presence of insulin. These plots also point out the high non-linearity between 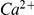 levels and secretion in islets, as found for individual cells (Figure 5). We also notice an islet regularity, due to glucose or insulin, which contrasts with cell to cell variability.

## Discussion

In this study, we have developed a simple model that quantitatively reproduces the rates of glucagon secretion by 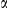-cells undergoing glucose-induced 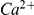 changes. We found that, although 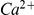 oscillations are highly variable, the average secretion rates predicted by the model fall into the range of values reported in the literature both for high and low glucose. Moreover, the model leads to interesting hypotheses concerning the molecular mechanisms that govern and regulate glucagon release. In particular, the model predicts that, in contrast to chromaffin cells, just one type of releasable granules could manage glucose-induced glucagon secretion in 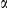-cells. Besides that, the secretory process is very similar in both cell types, involving three steps of 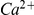 binding, with similar affinities. In agreement with this, a low affinity 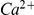 -sensing protein (known as synaptotagmin) mediates the fusion of the secretory granules to the plasma membrane in both cell types [7], [22]. This is similar to pancreatic 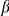-cells as well [23].

Our modeling approach, however, emphasizes the importance of a specific regulation of secretion in 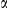-cells. The model indeed takes into account that the resupply of releasable granules is not only controlled by cytoplasmic calcium, as in neuroendocrine and 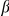-cells, but also by the level of extracellular glucose [26]. Here, we assume that resupply rate has a linear dependence on glucose concentration (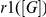), which leads to a good agreement for both secretion rate and granule distribution at high and low glucose concentrations. Taking into account the maximal resupply rate estimated for mouse 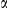-cells under depolarization-induced exocytosis (20 granules per second) [19], the model indeed predicts the observed steady-state secretion rate at low glucose reported in [7] (i.e. 0.8 to 1 pg/islet/minute). Moreover, the proposed linear function for glucose sensitivity of resupply also leads to a good estimation of granule mobilization for stimulated (low-glucose) conditions, as the resupply rate is increased about 2.5-fold for high glucose (up to 51.5 granules per second), which agrees with the 3.3-fold reported in [26] although this was observed for rat 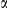-cells.

Our results indicate that glucose may regulate glucagon secretion through two different and counter-acting actions in 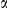-cells. On the one hand, low glucose stimulates calcium oscillations that induce and potentiate glucagon release. On the other hand, low glucose reduces the rate of granule resupply. Our physiological interpretation of this apparent paradox is that during low glucose periods, there would be a well-controlled number of releasable granules refilled slowly from a huge reserve pool to ensure a steady-state secretion rate that could last for several minutes (as observed by [7]). This would be reasonable to avoid that 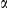-cells would exhaust the releasable pool of granules, and could mean also that the 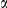-cell has its own glucose-sensing mechanisms as discussed in some works [1], [9].

About the effect of insulin in islets at low glucose, we have observed a reduction of the mean 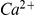 level in each islet (Figure 7(A)), which is responsible for the decrease in glucagon secretion predicted by the model (Figure 7(B)). With the insulin concentration used in the present study (4 nM) at low glucose, insulin-induced reduction of secretion is less efficient than the inhibition induced by high glucose. In the pancreas, glucose has an inhibitory effect by itself but at the same time, it can activate other inhibitory processes from 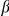-cells such as insulin secretion [11]. Our study suggests that insulin and glucose both reduce glucagon release by lowering intracellular 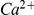 in 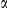-cells. Therefore, we cannot exclude that in experiments performed on intact islets, a high concentration of glucose in the extracellular solution can induce some insulin secretion; glucose elevation may thus modulate glucagon secretion both directly and through insulin, leading to a stronger inhibition.

Overall, the present results emphasize the main role played by 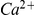 and glucose in the control of glucagon secretion by 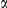-cells. Glucose control is occurring both via a direct pathway that affects the resupply of secretory granules and via a control of 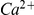 dynamics, while insulin is only modulating intracellular 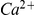 dynamics. The cellular mechanisms and specific pathways involved in both regulations need to be unveiled. As other previous studies [33], [35], our modeling approach of glucose-induced glucagon secretion in 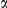-cells also enlightens the potentiating role of 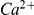 oscillations versus constant levels with the same average, as summarized in Table 3. Thus, at low glucose concentrations, whole-islet glucagon secretion may be elevated not only by a higher average 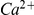 in each 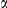-cell, but also by the release increment induced by 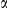-cells exhibiting 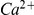 oscillations. Such an observation suggests new therapeutic targets as the level of secretion might be manipulated by drugs affecting both the levels and the dynamics of 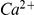 increases.

We conclude that under normal functioning, the 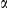-cell would be able to respond for long hypoglycemic periods as speculated in some works [28], but under pathological conditions as in diabetic patients, it may be unable to maintain the constant secretion rate probably due to abnormal blood glucose levels that would affect granule mobilization, and would induce insulin secretion leading to reduced islet 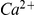 levels. In the near future, this model could also be implemented to simulate and/or predict calcium signals and glucagon release by pancreatic 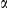-cells in pathological situations such as type 1 diabetes, which is characterized by a lack or very low concentrations of insulin and elevated glucose levels. Other pathological situations, such as insulin resistance and hyperinsulinemia that take place during the onset of type 2 diabetes could be also modeled.
